# Peer Review of “Satisfaction With Health Care Services at the Pediatric Specialist Clinic of the National Referral Center in Malaysia: Cross-sectional Study of Caregivers’ Perspectives”

**DOI:** 10.2196/37050

**Published:** 2022-05-25

**Keywords:** pediatrics, caregivers, health care services, public hospital, Malaysia, public-private-partnership, children

*This is a peer-review report submitted for the paper “Satisfaction With Health Care Services at the Pediatric Specialist Clinic of the National Referral Center in Malaysia: Cross-sectional Study of Caregivers’ Perspectives."*

## Round 1 Review

### General Comments

Thanks for the opportunity to review this manuscript [1] entitled “Caregivers’ Perspective—Satisfaction With Healthcare Services at the Paediatric Specialist Clinic of the National Referral Centre in Malaysia.” The authors report on an important topic, and their research work will contribute to the existing literature. Overall, the manuscript is well written with enough details in different sections. The tables are informative. The following are comments/concerns for the authors to consider.

### Specific Comments

- Abstract: include data/numbers in the Results section rather than general summary statements
- Introduction: include any a priori hypotheses
- Introduction: to support the rationale for the review, the authors should include additional recent promising evidence that supports the feasibility, acceptability, and efficacy of digital health interventions in different chronic medical conditions to provide context for the applicability of lessons learned in the study across other fields [2-7].
- Discussion: two recent reviews focused on pediatric/adolescent care and COVID-19 with mobile health (mHealth)/eHealth and adolescent/children psychosocial well-being, both worth discussing [8,9]
- Discussion: the authors could consider including a paragraph on study strengths.
- Discussion: it is critical to discuss the value of including direct patient input in the development of mHealth interventions, and other key considerations for end users should be sought early on in the process of app or digital health intervention design to ensure long- and short-term engagement [10-13].
- Discussion: the authors should expand and elaborate more on how their findings support or contrast available literature and provide suggestions for future research directions that would address existing knowledge gaps.
- Discussion: the authors should also acknowledge the lack of economic data to support the use of digital health interventions to date [14,15].

## Round 2 Review

### General Comments

No additional comments.

## Abbreviations

mHealth: mobile health
